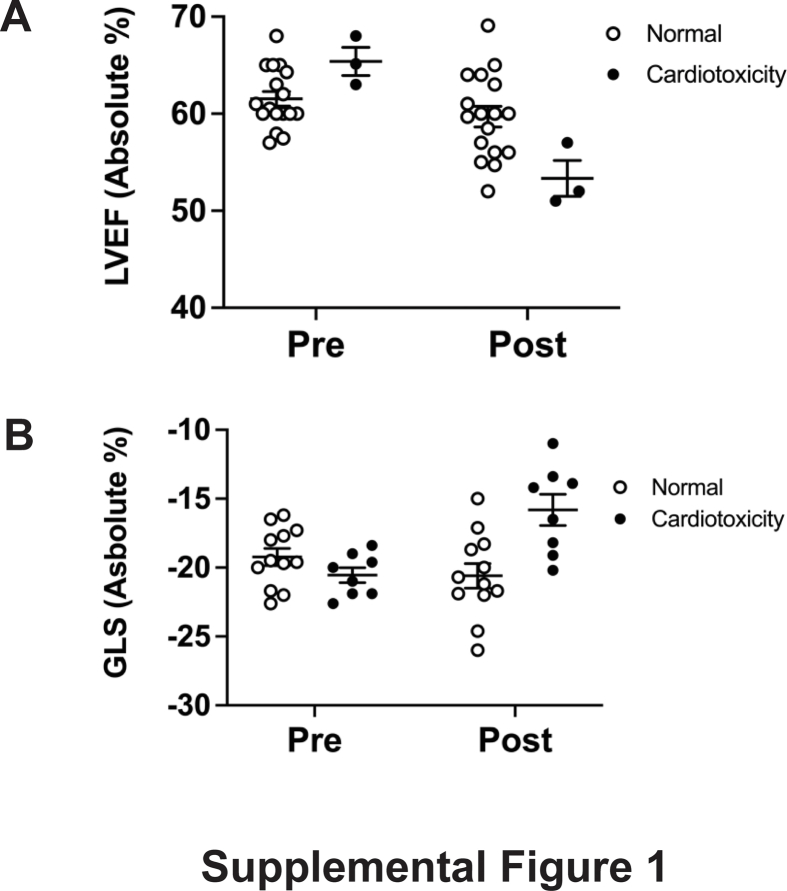# Serum and plasma sphingolipids as biomarkers of chemotherapy-induced cardiotoxicity in female patients with breast cancer

**DOI:** 10.1016/j.jlr.2025.100798

**Published:** 2025-04-05

**Authors:** Samia Mohammed, Andreas P. Kalogeropoulos, Victoria Alvarado, Michelle Weisfelner-Bloom, Christopher J. Clarke

**Affiliations:** 1 Department of Medicine, Stony Brook University, Stony Brook, NY, USA; 2 Cancer Center, Stony Brook University, Stony Brook, NY, USA; 3 Division of Cardiology, Department of Medicine, Stony Brook University, Stony Brook, NY, USA

**Keywords:** biomarkers, cardiotoxicity, doxorubicin, sphingolipids

## Abstract

Although effective as a chemotherapeutic, the utility of Doxorubicin (Dox) is hampered by cardiotoxicity. Despite this, the ability to predict and guide monitoring of patients receiving Dox is hampered by a lack of effective biomarkers to identify susceptible patients and detect early signs of subclinical cardiotoxicity. Based on their well-established roles in the response to Dox and other chemotherapies, we performed a retrospective analysis of serum and plasma sphingolipids (SLs) from female patients with breast cancer (BC) undergoing anthracycline-containing therapy, correlating with cardiac parameters assessed by echocardiography. Results showed substantial changes in both plasma and serum SL species during therapy including ceramide (Cer), deoxydihydroCer, and dihydrosphingosine with reversion toward baseline after treatment. Linear mixed-effects model analysis revealed that baseline levels of a number of SLs correlated with adverse cardiac outcomes. Here, serum sphingosine-1-phosphate (S1P), dihydroS1P, and plasma Cer performed comparably to the prognostic value of pro-NT-BNP, an established biomarker of cardiotoxicity. Intriguingly, while pro-NT-BNP had no predictive value at mid- and post-therapy timepoints, serum S1P and dihydroS1P, and plasma Cer levels showed a correlation with adverse outcomes, particularly at the post-therapy timepoint. Finally, analysis of plasma and serum C16:C24-Cer ratios—previously linked with adverse cardiac outcomes—showed no correlation in the context of chemotherapy treatment. Overall, this pilot study provides initial evidence that plasma and serum SLs may have benefits as both prognostic and diagnostic biomarkers for female BC patients undergoing anthracycline-containing chemotherapy. Consequently, diagnostic SL measurements—recently implemented for metabolic-associated cardiac disorders—could have wider utility.

Chemotherapeutics are a major component of cancer treatment and while effective at attacking cancer cells, they often come with associated side effects and toxicities. A major example of this relates to Doxorubicin (Dox) and other anthracyclines, which are used for treating many cancers and whose clinical utility is limited by dose-dependent cardiotoxicity (1, 2, 3). Although reports vary, dose-dependent cardiomyopathy has been reported to range from 7% at 150 mg/m^2^ to up to 65% at 550 mg/m^2^. Dox-induced cardiotoxicity presents as a spectrum of left ventricular dysfunction and clinical heart failure (1, 2, 3). Cardiotoxicity commonly occurs within 12 months of initial treatment but can also present after a significant delay (4, 5). The cardiac damage associated with Dox treatment is considered largely irreversible and can profoundly impact the survival and prognosis of cancer patients (1, 2, 3, 6, 7). Consequently, as cancer survival rates improve and there are increasing number of patients exposed to anthracyclines, strategies that can better predict and control this cardiotoxicity are crucial.

Echocardiography is currently the standard of care for monitoring cardiac function in patients with cancer, owing to widespread availability and safety. The addition of contrast-enhanced and three-dimensional echocardiography has improved subclinical detection of LV dysfunction (LVD) (8). Global longitudinal strain (GLS), a measure of myocardial contractility, provides additional predictive value for LVEF decline in patients receiving anthracyclines (9, 10). As such, the European Society of Cardiology Guidelines and recent cardiac imaging guidelines recommend the addition of transthoracic echocardiography (TTE) with GLS as part of the cardiac imaging assessment of patients receiving anthracycline-based chemotherapy (11). Nonetheless, despite improvements in imaging technology, there is still limited data to inform appropriate timing and interval for surveillance imaging prior to, during, and following anthracycline therapy. Additionally, while imaging can detect early signs of damage, there are still limited means for defining patient populations with higher susceptibility or predisposition to anthracycline toxicity. The use of serum or plasma biomarkers holds significant potential, both for the identification of at-risk patient populations as well as for monitoring of cardiotoxicity during treatment. Current biomarkers for anthracycline-induced cardiotoxicity include cardiac troponins (TnI, TnT) and N-terminal pro-BNP peptide (NT-BNP) with increased levels predictive of future LVD (12, 13, 14, 15, 16, 17). Some studies have also suggested these biomarkers can have negative predictive value with the absence of changes in their levels reflecting minimal changes in cardiac function (16, 17). Despite this, both biomarkers have limited and ill-defined roles in the prediction of subclinical cardiotoxicity, and significant changes in the levels of each biomarker may only be detected after cardiac damage has already occurred (12, 13, 14, 15, 16, 17). Consequently, novel biomarkers might add additional value in risk prediction.

Sphingolipids (SL) such as ceramide (Cer), sphingosine (Sph), and Sph-1-phosphate (S1P) are a family of bioactive lipids implicated in many cellular processes (18, 19). Cellular SL levels are controlled by an interlinked network of metabolic enzymes, and alterations in SL metabolism are widely associated with the cellular response to numerous chemotherapies across diverse cancers (20, 21, 22, 23, 24, 25, 26). Pathologically, dysregulation of SL metabolism and signaling is linked with a host of diseases including cancers, neurological diseases, and metabolic syndromes with some reports showing that levels of SLs and SL enzymes can correlate with disease prognosis (18, 19, 27, 28, 29). In addition to their cellular functions, many SL species are readily detected in serum and plasma. In this context, S1P is very well studied, complexing with HDL and ApoM and being able to influence signaling and functions of endothelial cells through binding to extracellular S1P receptors (30, 31). There is also growing evidence that variations in serum and plasma SL levels are associated with pathological conditions including atherosclerosis (32), sepsis (33), metabolic syndrome (34), and age-related macular degeneration (35). More recently, serum SL profiles were reported to predict asymptomatic versus symptomatic COVID-19 (36) while an independent study found that serum SLs were prognostic for COVID disease severity (37). In the context of heart disease and function, studies have established specific Cer species as diagnostic indicators of adverse cardiac events. Research by Petersen and colleagues has reported the predictive value of the ratio of C16-Cer to C24-Cer with a high ratio being associated with increased HF risk and altered LVEF (38, 39). Independently, researchers at the Mayo Clinic reported that a ‘ceramide score’ obtained through measurements of C16, C18, C24, and C24:1-Cer was a better predictor of future adverse cardiovascular events than monitoring cholesterol levels (40, 41) and led to the implementation of Cer measurements (the CERAM test) as a diagnostic tool. A similar approach by researchers in Utah found that an inclusive score of specific Cer, dhCer, and SM species was a better predictor of coronary artery disease than cholesterol levels (42). However, despite the central role that SLs play in the chemotherapy response, the effects of anthracyclines on serum and plasma SLs or their association with heart function have yet to be fully investigated.

In this study, we performed a retrospective sphingolipidomic analysis of plasma and serum samples from a pilot cohort of patients with breast cancer receiving anthracycline therapy. We analyzed changes in SL levels at baseline, mid, post, and 6-months post-treatment, and correlated lipid levels with subclinical changes in heart function detected by TTE and speckle tracking.

## Materials and Methods

### Patient samples

This study is a retrospective analysis of plasma and serum samples obtained from a previous prospective study into potential novel biomarkers of anthracycline-induced cardiotoxicity (43). In the prior study, serial blood and echocardiographic assessment was performed on female patients 18–85 years old with a diagnosis of invasive breast cancer without metastases who were planned for anthracycline-inclusive chemotherapy. Guidelines from the original study indicate that patients were excluded if they had: 1) a history of major heart disease at the time of cancer diagnosis; 2) a history of known coronary artery disease; 3) a history of clinical HF or previous hospitalization due to HF; 4) elevated levels of NT-proBNP (within 2 times the upper limit of normal) during baseline screening; 5) known history of other chemotherapy-treated malignancy. All recruited patients in the original study (31 total) received dose-dense Dox therapy (60 mg/m^2^) and cyclophosphamide (600 mg/m^2^). Twenty nine patients also received a taxane (paclitaxel) and 4 patients also received the targeted HER2 therapy trastuzamab. Full details are described in the prior study (43). Patients were assessed at baseline, mid-therapy, post-therapy, and at 6-month follow-up. Of the 31 patients screened in the original study, serum samples from 20 patients and plasma samples from 18 patients were obtained for analysis. The Institutional Review Board of Stony Brook University approved both the original study (protocol #922042) and the retrospective analysis (protocol #2020-00116) prior to initiation. The study protocol and procedures conformed to the standards set by the latest revision of the Declaration of Helsinki.

### Sphingolipidomic analysis and normalization

Patient samples were kept in long-term storage at −80°C. For lipid analysis, serum and plasma samples were thawed on ice and lipids were extracted as described previously (25, 44, 45) using the method of Bielawski *et al.* (46, 47). Briefly, 100–200 μl of serum/plasma was diluted in 2 ml RPMI medium, and 50 μl internal standard mix was added. Samples were extracted with 2 ml extraction solvent (isopropanol: ethyl acetate 15:85 v/v), vortexed, and centrifuged (5 min, 2500 *g*). The upper organic phase (approx. 2 ml) was transferred to a fresh glass tube. The remaining lower phase was acidified with 100 μl formic acid (98%) and an additional 2 ml of extraction solvent was added. Samples were vortexed and centrifuged (5 min, 2500 *g*) and the upper organic phase (approx 2 ml) was removed and combined with the prior extract. The total extract was dried under nitrogen gas and resuspended in 150 μl of mobile phase B (1 mM ammonium formate, 0.2% formic acid in methanol) and lipids were analyzed by tandem LC/MS/MS at the Stony Brook lipidomics core. Samples were analyzed for ceramide (Cer), dihydroCer (dhCer), sphingosine (Sph), Sph-1-phosphate (S1P), dihydroSph (dhSph), dhSph-1-phosphate (dhS1P), deoxydhCer, and alpha-hydroxyCer (ahCer). It should be noted that plasma or serum sphingomyelin (SM) levels were not assessed, primarily as prior studies have established SM as the major dominant species in both at >85% of lipid content (45) and based on experience in cell studies, we considered that we would be less likely to detect changes in SM mass. Additionally, all of the species that we assessed were those with the classical 18-carbon sphingoid backbone, and not with the 16-carbon backbone. Measured lipid levels were normalized to ml of plasma/serum. Internal standards (ITSD) used for analyses comprised varying mixes of 17C-Sph, 17C-dhSph, 17C-S1P, 17C-C16-Cer, 13C-C16-Cer, 17C-C18-Cer, 17C-C24:1Cer, and 17C-C24-Cer. For each analysis, the ITSD response was analyzed and consistency across samples was confirmed with ITSD levels used to normalize the signals using the closed structure analyte-internal standard. In addition, calibration and quality control samples were analyzed in parallel to each experiment. In all cases, samples with low recovery of internal standards or analytes outside the linearity of the calibration curve were not quantified.

### Statistical analysis

Continuous variables were described as a median (interquartile range) to ensure valid measures of location and dispersion regardless of distribution, and discrete (binary and categorical) variables were described as frequency (percentage). To examine changes over time for the exposures of interest (lipid species concentrations), and the association with outcomes of interest (echocardiographic variables), we employed linear mixed-effects models with patient intercept as the random effect (ie, each patient had an individual intercept for each variable of interest). Because of the small sample size, we estimated standard errors and confidence intervals for each model using bootstrapping (1,000 replications). Changes between time points for each variable of interest were estimated with appropriate contrasts for the marginal means after estimating the corresponding mixed-effects model. We used unadjusted α = 0.05 as the threshold for statistical significance. Analyses were performed with STATA 18.5 (StataCorp LLC, College Station, TX).

## Results

### Anthracycline-containing therapy is associated with alterations in plasma and serum SL levels

**Fig. 1:**
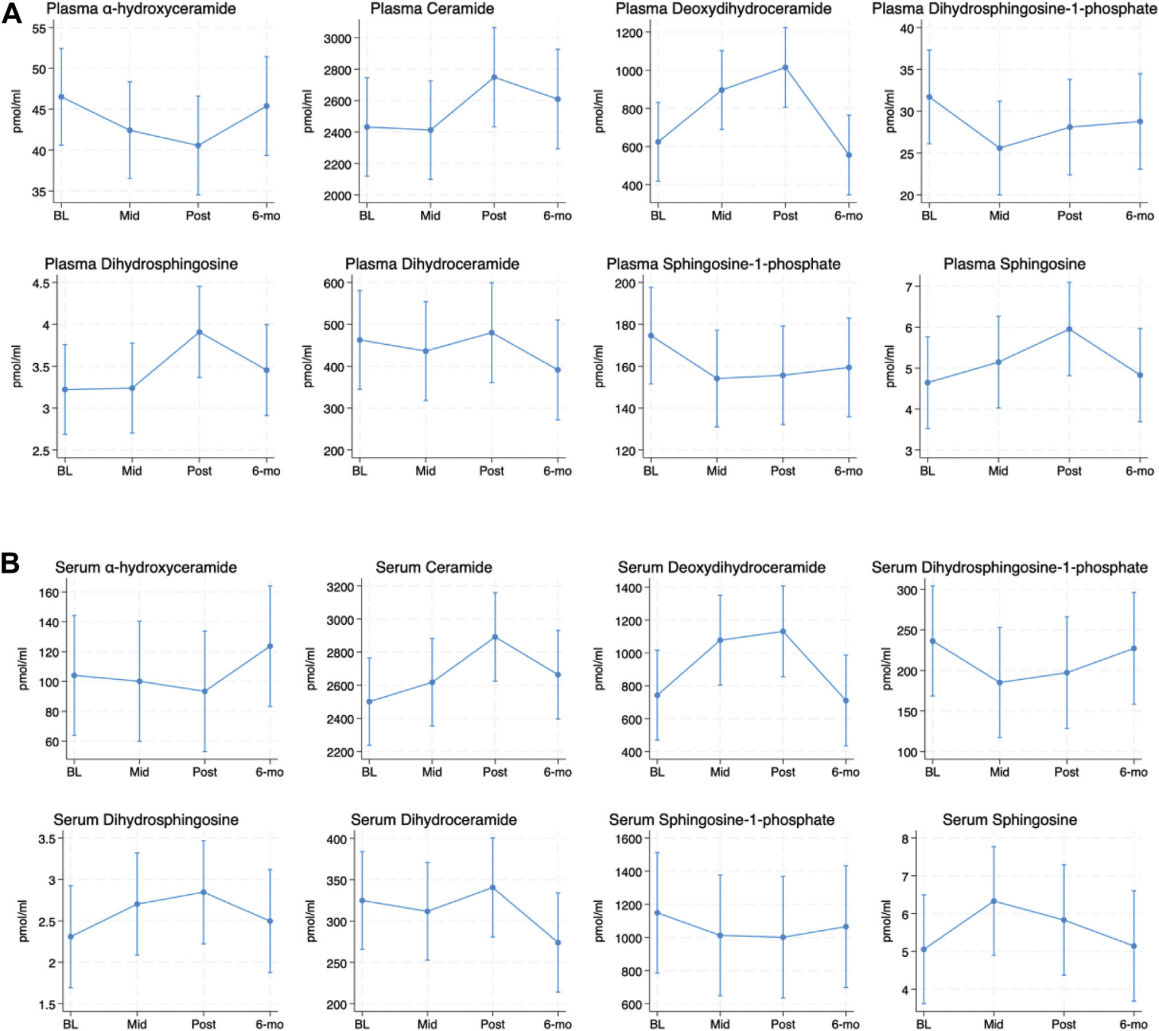
Alterations in plasma and serum sphingolipid levels in patients undergoing anthracycline-containing therapy. Lipids were extracted from 100-200ul of (A) plasma; and (B) serum, and analyzed for the sphingolipid classes shown as described in “Materials and Methods”. Data were normalized to ml plasma/serum and are expressed as pmol/ml at baseline (BL), midpoint (Mid), Post-therapy (post) and 6-months follow up (6-months). Data are expressed as mean ± 95% confidence interval.

Changes in cellular SL levels are a well-established hallmark of the response to many chemotherapies (20, 21, 22, 23, 24, 25, 26). In contrast, few studies have examined the effects of chemotherapies on SLs in normal tissue, nor in patient samples. Here, we performed a retrospective sphingolipidomic analysis of a cohort of plasma (18 total) and serum samples (20 total) collected at baseline, mid-therapy, end-therapy, and at 6-month follow-up from breast cancer patients undergoing anthracycline therapy. Lipid profiles for plasma (Fig. 1A) and serum (Fig. 1B) are summarized. Of note, highly similar profiles were observed between serum and plasma for all lipids analyzed with the primary difference being in the absolute levels of the lipids. In this context, plasma samples tended to have lower absolute levels for all lipids with the exceptions of dhCer and dhSph, which were higher in plasma than serum. For Cer, deoxydhCer, and dhSph, levels in both plasma and serum increased throughout therapy, peaking at the post-therapy time point and decreasing towards baseline at follow-up. For S1P and dhS1P, levels in both plasma and serum tended to decrease with therapy and remain somewhat flat throughout post-therapy and follow-up. Similar decreases with therapy were observed with both plasma and serum ahCer but at follow-up, levels had begun to increase toward the baseline. For dhCer, levels remained somewhat flat throughout therapy in both plasma and serum but sharply decreased at 6 months follow-up. Finally, levels of Sph tended to increase with therapy and return towards baseline at follow-up, but there were differences as to the peak levels—with serum Sph peaking in mid-therapy and plasma Sph peaking at follow-up. Given the small sample size, the confidence interval for lipid values is broad but there were nonetheless some statistically significant changes observed. In serum, there were significant changes in deoxydhCer at the midpoint (*P* = 0.0034) and post-therapy (*P* = 0.0045) versus baseline levels, Cer at post-therapy versus baseline (*P* = 0.0005), and dhSph at post-therapy versus baseline (*P* = 0.016). In plasma, there were similar significant changes in deoxydhCer at midpoint (*P* = 0.0087) and post-therapy (*P* = 0.0008) versus baseline levels, Cer at post-therapy versus baseline (*P* = 0.0099), and dhSph at post-therapy versus baseline (*P* = 0.0202). There were also significant changes in dhS1P at mid-therapy versus baseline (*P* = 0.0142) and Sph at post-therapy versus baseline (*P* = 0.0229). Taken together, these results show that alterations in serum and plasma SL levels are associated with anthracycline-containing therapy regimens.

### Correlation of baseline SL levels with adverse changes in cardiac function (LVEF, GLS)

Prior studies have associated circulating levels of SLs (mainly Cer) with an increased likelihood of HF and adverse cardiovascular outcomes (36, 37, 38, 39, 40). Accordingly, we reasoned that baseline SL levels may be associated with an increased susceptibility to Dox-induced cardiac damage. Here, it is important to note that adverse cardiac function is reflected by a decrease in LVEF and/or an increase in GLS (eg −20% to −15%). Consequently, if elevated levels of lipids are associated with adverse heart function, then we would expect a negative correlation with LVEF and a positive correlation with GLS. As detailed in the original study (43) and based on clinical guidelines, cardiotoxicity was defined as either: 1) A greater than or equal to 10% reduction in absolute LVEF (ie a change of 70%–60%); or 2) A greater than or equal to 10% reduction from baseline in GLS (eg −20% to −18% is 2% absolute change but 10% change from baseline). When considering these criteria, cardiotoxicity was present at 6-month follow-up in 3 patients from changes in LVEF (Supplemental Fig. S1A, **filled circles**) and 8 patients from changes in GLS (Supplemental Fig. S1B, **filled circles**). Of these, 2 patients exhibited changes in both parameters. Overall, of the samples analyzed for serum, 9/20 patients met the defined criteria for cardiotoxicity at follow-up, while for plasma, 8/18 patients met the defined criteria for cardiotoxicity at follow-up.

**Fig. 2:**
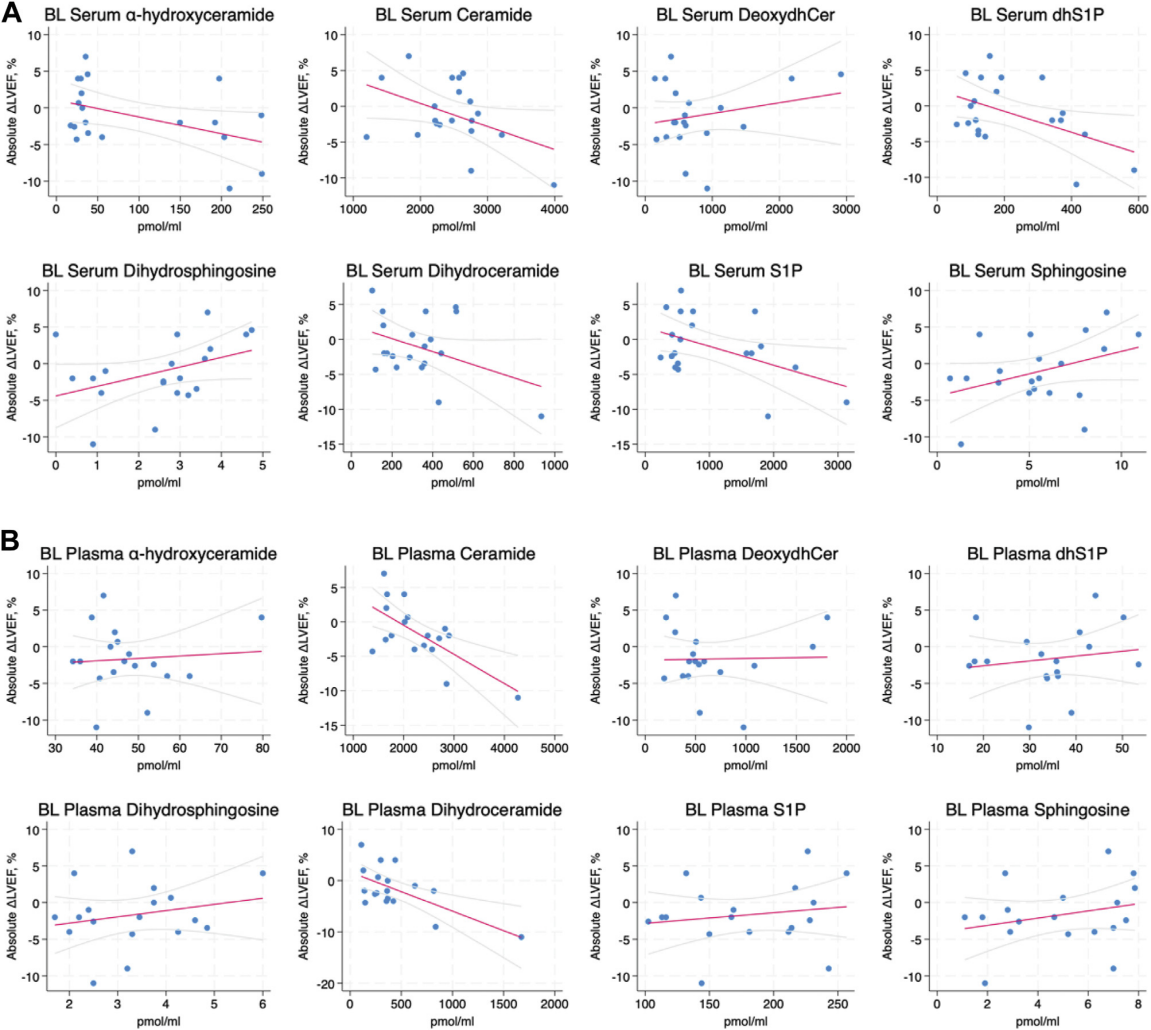
Baseline plasma and serum sphingolipid levels correlated with changes in LVEF in patients receiving anthracycline-containing therapy. Linear mixed-effects models were used to correlate baseline lipid levels for (A) serum and (B) plasma with changes in LVEF from baseline to 6 months follow up. Here, a decrease in LVEF would be considered an adverse outcome. Trendline is shown in red with 95% confidence interval shown in light gray. Abbreviations: Deoxydhcer = deoxydihydroceramide; dhS1P = dihydrosphingosine 1-phosphate; S1P = sphingosine 1-phosphate.

**Fig. 3:**
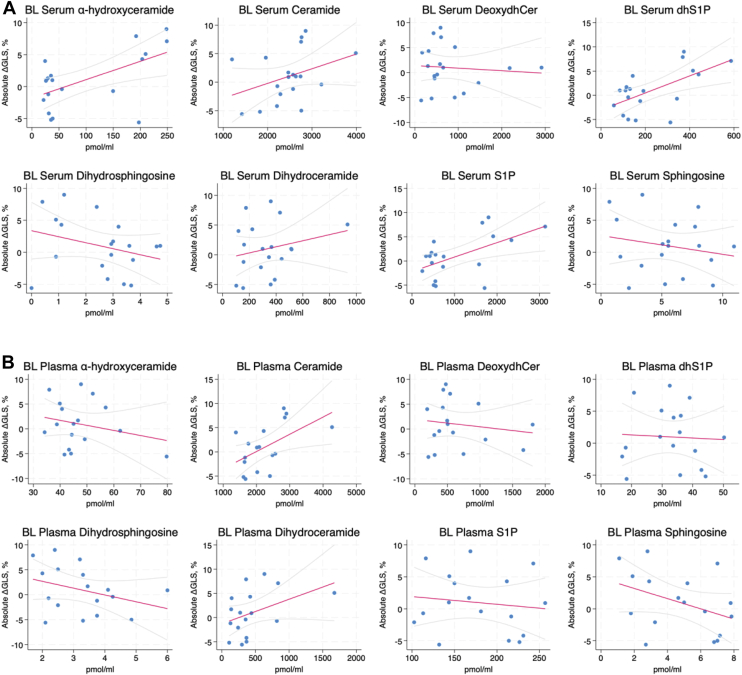
Baseline plasma and serum sphingolipid levels correlated with changes in GLS in patients receiving anthracycline-containing therapy. Linear mixed-effects models were used to correlate baseline lipid levels for (A) serum and (B) plasma with changes in GLS from baseline to 6-month follow-up. Here, an increase in GLS would be considered an adverse outcome. The trendline is shown in red with 95% confidence interval shown in light gray. Abbreviations: Deoxydhcer = deoxydihydroceramide; dhS1P = dihydrosphingosine 1-phosphate; S1P = sphingosine 1-phosphate.

To correlate baseline SL levels in serum or plasma with subsequent changes in LVEF and GLS, we employed linear mixed models analysis. With LVEF (Fig. 2), there were significant negative correlations with baseline serum levels of S1P (r = −0.485, *P* = 0.038) and dihydroS1P (r = −0.489, *P* = 0.034) with a negative, but marginally insignificant correlation observed for ahCer (r = −0.449, *P* = 0.067). In contrast, there were negative correlations of LVEF with baseline plasma levels of Cer (r = −0.671, *P* = 0.002) and dhCer (r = −0.652, *P* = 0.008). Analysis of baseline lipid levels with changes in GLS (Fig. 3) found significant correlations with serum S1P (r = 0.555, *P* = 0.007) and dihydroS1P (r = 0.601, *P* = 0.001) consistent with LVEF results. Notably, here there were also significant correlations with ahCer (r = 0.580, *P* = 0.011). For baseline plasma levels, there were positive correlations of GLS changes with Cer (r = 0.537, *P* = 0.046), consistent with LVEF correlations, but there was no significant correlation of GLS with dhCer levels (r = 0.415, *P* = 0.168). To provide a point of comparison, we correlated levels of pro-NT-BNP—an established biomarker of anthracycline cardiotoxicity that was previously investigated in this cohort (43)—with subsequent changes in LVEF and GLS from baseline to post-therapy and 6 months follow up. This analysis revealed that baseline pro-NT-BNP levels negatively correlated with LVEF changes but this was not quite statistically significant (r = −0.469, *P* = 0.082). However, there was a significant correlation between pro-NT-BNP and GLS changes (r = 0.596, *P* = 0.001) (Table 1). Taken together, these results suggest that both plasma and serum SL levels can be predictive of anthracycline-driven changes in LVEF and GLS, and that they function comparably to an existing biomarker.

**Table 1:** Correlation of the established biomarker pro-NT-BNP with cardiac parameters at baseline, mid-therapy, and post-therapy timepoints.

	LVEF		GLS	
	Beta	*P*-value	Beta	*P*-value
Baseline	−0.469	0.082	0.596	0.001
Mid-therapy	0.014	0.956	0.156	0.510
Post	−0.127	0.660	0.282	0.267

Shown are corresponding beta value and *P*-value for correlation of pro-NT-BNP at the respective timepoints with changes in LVEF and GLS as shown.

### Correlation of mid and post-therapy SL levels with adverse changes in cardiac function (LVEF, GLS)

Prognostic biomarkers can be informative for identifying those patients who may have an increased likelihood of anthracycline-induced cardiotoxicity. However, an early indicator of subclinical cardiotoxicity would also have value as this could provide guidance for monitoring of cardiac function, administering drugs that can try to preserve heart function, or dose reduction of anthracyclines. For similar reasons, a post-therapy biomarker that is predictive of subsequent adverse cardiac changes would also have value. Notably, in this patient cohort, while pro-NT-BNP levels had been previously observed to increase during treatment (43), mid-therapy levels of pro-NT-BNP were not predictive of subsequent changes for LVEF (r = 0.014, *P* = 0.956) or GLS (r = 0.156, *P* = 0.510). Similarly, post-therapy levels of pro-NT-BNP did not correlate well with subsequent changes in LVEF (r = −0.127, *P* = 0.660) or GLS (r = 0.282, *P* = 0.267) although results were marginally better for GLS than for LVEF. As the correlation of baseline SL levels with adverse LVEF and GLS outcomes showed promise and changes in both plasma and serum SL levels during therapy were observed, we hypothesized that midpoint and post-therapy SLs might also have predictive value for LVEF and GLS changes.

Analysis of serum SL levels (Table 2) with LVEF revealed no significant correlations with midpoint lipid values—although ahCer (r = −0.414, *P* = 0.064) was borderline. However, at the post-chemotherapy endpoint, there were significant negative correlations with serum S1P levels and LVEF (r = −0.488, *P* = 0.016) with ahCer again showing borderline significant (r = −0.425, *P* = 0.054). In contrast, comparisons of serum SL levels with GLS showed more promising results; at midpoint, there were significant correlations of S1P (r = 0.570, *P* = 0.005), dihydroS1P (r = 0.569, *P* = 0.007) and ahCer (r = 0.583, *P* = 0.020) although at the post-chemotherapy time-point, there was only significant correlations with S1P (r = 0.500, *P* = 0.034) and, again, borderline significance for ahCer (r = 0.486, *P* = 0.053). For plasma SL levels (Table 3), comparisons with LVEF revealed no significant negative correlations with midpoint lipid values – although intriguingly there were positive correlations (although not quite statistically significant) for dhSph (r = 0.478, *P* = 0.083) and dhS1P (r = 0.444, *P* = 0.088). However, at the post-chemotherapy endpoint, there was a significant negative correlation of LVEF with plasma Cer (r = −0.527, *P* = 0.019). Comparison of plasma SL levels with GLS also showed no positive correlations at midpoint although surprisingly, there were significant negative correlations with Sph (r = −0.448, *P* = 0.043) and dhSph (r = −0.550, *P* = 0.005). However, at the endpoint, there was a significant positive correlation with Cer (r = 0.516, *P* = 0.047) and a negative correlation with dhS1P (r = −0.483, *P* = 0.041). Taken together, these results suggest that plasma and serum SLs can have prognostic value for adverse cardiac outcomes both during and immediately post-therapy—although results were more robust with GLS than LVEF. They also suggest that some SLs could also be positive indicators of cardiac function during anthracycline therapy.

**Table 2:** Correlation of serum sphingolipid levels with cardiac parameters at mid-therapy and post-therapy time points.

	Mid LVEF		Mid GLS		Post LVEF		Post GLS	
	Beta	*P*-value	Beta	*P*-value	Beta	*P*-value	Beta	*P*-value
Cer	−0.160	0.368	0.131	0.597	−0.330	0.177	0.254	0.238
dhCer	0.095	0.679	0.164	0.463	−0.044	0.886	0.105	0.632
S1P	−0.317	0.149	0.570	0.005	−0.488	0.016	0.500	0.034
dhS1P	−0.190	0.274	0.569	0.007	−0.438	0.100	0.439	0.177
Sph	0.273	0.302	−0.246	0.272	−0.191	0.535	0.094	0.741
dhSp	0.372	0.134	−0.341	0.124	0.044	0.872	−0.124	0.665
deoxydhCer	0.283	0.245	−0.138	0.514	0.229	0.348	−0.077	0.732
ahCer	−0.414	0.064	0.583	0.020	−0.425	0.054	0.486	0.053

Shown are the corresponding beta value and *P*-value for the correlation of serum sphingolipid levels at mid- and post-therapy timepoints with subsequent changes in LVEF and GLS.

**Table 3:** Correlation of plasma sphingolipid levels with cardiac parameters at mid-therapy and post-therapy time points.

	Mid LVEF		Mid GLS		Post LVEF		Post GLS	
	Beta	*P*-value	Beta	*P*-value	Beta	*P*-value	Beta	*P*-value
Cer	−0.181	0.392	0.339	0.220	−0.527	0.019	0.516	0.047
dhCer	−0.163	0.388	0.427	0.110	−0.367	0.152	0.348	0.133
S1P	0.243	0.333	0.006	0.986	0.214	0.439	−0.367	0.151
dhS1P	0.444	0.088	−0.165	0.541	0.384	0.142	−0.483	0.041
Sph	0.457	0.153	−0.448	0.043	0.075	0.817	−0.303	0.356
dhSp	0.478	0.083	−0.550	0.005	0.086	0.763	−0.317	0.331
deoxydhCer	0.240	0.380	−0.152	0.533	0.190	0.549	−0.060	0.841
ahCer	−0.181	0.512	0.192	0.488	−0.097	0.687	0.090	0.779

Shown are the corresponding beta value and *P*-value for the correlation of plasma sphingolipid levels at mid- and post-therapy time points with subsequent changes in LVEF and GLS.

### The C16:C24-Cer ratio does not correlate with adverse changes in cardiac function in anthracycline patients

Prior research has reported that the ratio of plasma C16-Cer to C24-Cer is a predictor of adverse cardiac outcomes superior to cholesterol levels (38), with follow-up studies suggesting that a higher C16: C24 ratio was associated with lower LVEF and lower global circumferential strain but was not associated with changes in GLS (39). Given the results above, we were curious if the C16: C24-Cer ratio may be similarly predictive of Dox-induced cardiotoxicity and assessed the correlation of serum and plasma C16:C24-Cer at baseline, mid-therapy, and post-therapy with LVEF and GLS as above (Table 4). Analysis of the serum C16: C24-Cer ratio showed no significant correlation with changes in LVEF at baseline (r = −0.099, *P* = 0.798), midpoint (r = −0.271, *P* = 0.329), or post-chemotherapy (r = −0.436, *P* = 0.098) although it should be noted that the correlation was approaching significance at post-therapy. Comparison of serum C16: C24-Cer ratio with GLS showed similar trends with no significant correlations at baseline (r = 0.013, *P* = 0.978), midpoint (r = 0.275, *P* = 0.364), or post-chemotherapy (r = 0.287, *P* = 0.268) although in contrast with LVEF, there was less improvement at later time points. Analysis of plasma C16:C24-Cer ratios showed there were no significant correlations for changes in LVEF at baseline (r = 0.104, *P* = 0.719), midpoint (r = −0.175, *P* = 0.466), or post-chemotherapy (r = 0.052, *P* = 0.816). Similar results were obtained for changes in GLS at baseline (r = −0.046, *P* = 0.861), midpoint (r = 0.151, *P* = 0.526), or post-chemotherapy (r = −0.172, *P* = 0.485). Taken together, these results suggest that the C16:C24-Cer ratio—either in serum or plasma—is not predictive of the cardiotoxic effects of anthracycline therapy.

**Table 4:** Correlation of serum and plasma C16:C24-Cer ratio with cardiac parameters at baseline, mid-therapy, and post-therapy time points.

	LVEF		GLS	
	Beta	*P*-value	Beta	*P*-value
Serum				
Baseline	−0.099	0.798	0.013	0.978
Mid-therapy	−0.271	0.329	0.275	0.364
Post	−0.436	0.098	0.287	0.287
Plasma				
Baseline	0.104	0.719	−0.046	0.861
Mid-therapy	−0.175	0.466	0.151	0.526
Post	0.052	0.816	−0.172	0.551

Shown are the corresponding beta value and *P*-value for the correlation of serum and plasma C16:C24-Cer ration at the time points shown with subsequent changes in LVEF and GLS.

## Discussion

In this study, we performed a retrospective analysis of SL levels in serum and plasma samples from anthracycline-treated patients, correlating lipid levels with LVEF and GLS parameters of cardiac function. Our results show robust changes in plasma and serum SLs during anthracycline-containing regimens, with Cer, deoxydhCer, and dhSph being the most notable species affected. Furthermore, we found correlations between plasma and serum SLs at baseline, mid-therapy, and post-therapy with adverse changes in cardiac function. In contrast, the ratio of C16:C24-Cer, previously found to be a predictor of worse cardiac outcomes, was not predictive of anthracycline-induced effects. Taken together, this pilot study offers preliminary evidence in support of plasma and serum SLs as potential biomarkers of interest for anthracycline-induced cardiotoxicity.

Since the discovery of their signaling functions, the biological roles of SLs have been the subject of intensive research, and alterations in cellular SLs are now well-established components of the response to various chemotherapies (20, 21, 22, 23, 24, 25, 26). While this has led to interest in modulating SLs to enhance therapeutic efficacy in cancer cells, comparatively few studies have looked at the effects of chemotherapies on SLs in non-transformed tissues, nor in plasma or serum of chemotherapy-treated patients. Here, our results show that alterations in plasma and serum SL levels occur during anthracycline-containing therapy regimens. These changes were broad, occurring across multiple SL species, and despite being from a small cohort, some of these changes reached statistical significance—particularly for deoxydhCer, Cer, and dhSph in both plasma and serum. Lipid levels were also clearly driven by treatment as levels increased/decreased at mid-therapy and post-therapy time points before reverting towards the baseline levels at 6 months of follow-up. Thus, as with many other pathological conditions (32, 33, 34, 35, 36, 37), chemotherapy can drive systemic changes in plasma and serum SLs. Although levels were used to correlate with cardiac function, the source of these lipids is unclear as anthracyclines and other chemotherapeutics can also affect vascular endothelial cells (48, 49) and other tissues (50, 51) as well as the cancer cells themselves (21, 22). One potential way that this could be addressed would be to measure the levels of sphingolipids harboring a 16-carbon sphingoid base—rather than the conventional 18-carbon sphingoid base that we measured here. As recent studies have reported an elevated pool of C16-backbone sphingolipids in the heart owing to elevated levels of SPTLC3 (52, 53), it is possible that plasma and serum changes in these lipids may more accurately reflect direct actions of chemotherapeutics on the heart. In this regard, it should also be noted that the observed changes in sphingolipids seen cannot be attributed solely to anthracyclines as nearly all patients in the original study received both cyclophosphamides and taxanes in addition to doxorubicin (43). Indeed, taxanes have been reported to affect SL metabolism in vitro (25, 26) and carry a risk of cardiotoxicity, particularly when in combination with anthracyclines (54, 55). Moreover, SLs have been linked with taxane-induced peripheral neuropathy in patients with BC, although notably, the non-canonical 1-deoxysphingolipids were more relevant in this context (25). Given that deoxydhCer levels were increased in our studies (Fig. 1), this raises the question of whether changes in specific lipid species could be a consequence of distinct chemotherapeutic agents although it should be noted that patients in this prior study received anthracyclines and cyclophosphamide as well (25). Finally, both cyclophosphamide and trastuzumab can be cardiotoxic or may exacerbate the cardiotoxic effects of anthracyclines (56, 57). However, effects of either drug on SL metabolism have been less well studied and in the case of trastuzumab, were only a factor in a small number of patients from the original study (4 out of 29) (43). While pre-clinical i*n vivo* studies with single agents would be the cleanest way to answer such questions, the multiple therapies are important to keep in mind when considering the broader applicability of these findings to other patient populations.

While echocardiography remains the standard of care for cardiac monitoring, serum and plasma biomarkers hold significant potential for identifying at-risk populations prior to treatment as well as in the detection of subclinical toxicity during chemotherapy. Here, our results show that both plasma and serum levels of a number of SL species were correlated with subsequent decreases in cardiac function as indicated by decreased LVEF and GLS. Notably, correlations of baseline SL species with adverse outcomes were at least comparable to or better than the existing biomarker pro-NT-BNP at predicting adverse changes in GLS and LVEF (and NT-BNP was not quite statistically significant for the latter). However, at mid-therapy and post-therapy timepoints, the levels of SL species—primarily S1P, dhS1P, and ahCer in serum, and Cer in plasma—were better at predicting subsequent adverse GLS or LVEF changes; indeed, mid and post-therapy pro-NT-BNP levels in this cohort were not predictive for either parameter. While baseline correlations can provide guidance on patient monitoring, correlations during treatment can be equally valuable for indicating whether cardioprotective intervention should be initiated, particularly as studies have shown that the earlier the intervention, the more substantial the recovery of LV function (58, 59). As there is evidence that changes in GLS can be more indicative of early subclinical cardiotoxicity (9, 10), these correlations may be of more clinical relevance. In this context, the correlations of SL levels with adverse GLS changes were more robust than those with LVEF—particularly at the mid-therapy time-point. An additional point of interest here is that midpoint plasma levels of Sph and dhSph showed a negative correlation with GLS (Sph – r = −0.448; dhSph –r = −0.550) and positive correlations with LVEF (albeit not statistically significant). Similar correlations for GLS were also observed at post-therapy with plasma dhS1P levels (GLS - r = −0.483). As these correlations would be more indicative of maintained or improved cardiac function, this raises the intriguing possibility that serum or plasma SLs could function both as negative and positive indicators of cardiac function during chemotherapy. We also note that while our focus in this study was on total lipid levels, there is the possibility that individual lipid species from each family may have comparable or better predictive power and serve to drive the broader correlations seen with total lipids. Indeed, the Cer score from Mayo Clinic centers on C16, C18, C24, and C24:1-Cer species (40, 41). However, given the number of potential SL species that could be measured (both in serum and plasma), we considered this more complex analysis beyond the scope of this initial study. However, the lack of correlation of the C16:C24-Cer ratio with adverse outcomes suggests that chemotherapy effects on the heart are distinct from those associated with more general adverse cardiac health changes. Nonetheless, we do note that at post-chemotherapy time points, the serum C16: C24-Cer ratio showed a negative correlation with subsequent LVEF changes and was approaching significance. Thus, it is plausible that analysis of a larger patient cohort may bring these results in line with prior reports. Finally, it is also noteworthy that the associations of specific lipids with cardiac outcomes vary between serum and plasma—with S1P and dhS1P showing more relevance in serum, but Cer being more relevant in plasma. This could be a consequence of the higher S1P and dhS1P levels in serum allowing for a greater “signal to noise” ratio than in plasma. Although it is unclear why both lipids are relatively higher in serum versus plasma, these results are in line with the thorough evaluation of serum and plasma lipids conducted by Hammad *et al.* (45). This could also be a consequence of the collection method utilized as it was previously reported that the anticoagulant used for plasma collection can have some effects on lipid detection (45). As there was no information on the collection method used for these studies, this is a potentially confounding factor for our study. Finally, it is also possible that the differential correlations are a reflection of the smaller sample size in this study and that a larger sample size may have produced consistent correlations across plasma and serum. This and other limitations are discussed below.

### Study limitations

Although these results show promise for SLs as biomarkers, there are limitations of this study that are important to note. First and foremost, this was a pilot study and limited to 18 sets of plasma and 20 sets of serum; thus, the study does not have sufficient power to detect major changes in biomarkers, SL levels, or cardiac changes. An important aspect related to this is that the vast majority (c. 90%) of the obtained samples were from a Caucasian population and as well as being from patients with few cardiac risk factors; thus, the relevance of these findings to broader populations or to patients who may have an elevated susceptibility to cardiotoxicity is limited and unclear. Related to this, in addition to the small sample size, this study analyzed samples obtained from female breast cancer patients; thus, the applicability of this to male cancer patients—or, indeed, female patients with other cancers is unclear. Finally, samples and cardiac function data were limited to a 6-month follow-up period—and given that anthracycline-induced cardiotoxicity commonly occurs up to 12 months post-therapy (4, 5), a follow-up at later time points could have provided further insight—although it should be noted that approx. 45% of the patients in the analyzed samples displayed GLS or LVEF parameters consistent with cardiotoxicity at the 6-month follow-up time point. Nonetheless, a follow-up retrospective or future prospective analysis with a larger sample population that keeps these considerations in mind is crucial to extend and further validate these findings.

In conclusion, this study has shown that anthracycline-induced chemotherapy regimens are associated with alterations in plasma and serum SLs in female breast cancer patients and provides evidence that plasma and serum SLs have the potential to serve as biomarkers for chemotherapy-induced cardiotoxicity. Importantly, SL levels were correlated with adverse changes in both GLS and LVEF, performed at least comparably to the existing biomarker pro-NT-BNP, and showed promise both for pre-treatment identification and mid/post therapy detection of patients with increased likelihood of adverse cardiac outcomes. Although preliminary in nature, these results suggest that the use of clinical SL measurements as a diagnostic tool for cardiac function could be applied to patients undergoing treatment with anthracyclines or other known cardiotoxic chemotherapies. They may also be beneficial for guiding the monitoring and management of cardiac function both during and after therapy.

## Data availability

The data and mixed model analysis results described in the manuscript are available upon request.

## Supplemental data

This article contains supplemental data.

## Conflict of interest

The authors declare that they have no conflicts of interest with the contents of this article.

## Figures and Tables

**Supp Fig 1:**